# College and Hospital Notes

**Published:** 1904-01

**Authors:** 


### COLLEGE AND HOSPITAL NOTES.

It is announced that the Lackawanna Steel Company will erect a hospital and morgue at Stony Point for the treatment of the maimed and for the reception of the bodies of those killed in the service of the company. At present those injured are taken to the Buffalo General Hospital, which is regarded as inconvenient. The cost of the new structure, which will be built of wood, is expected to aggregate about $12,000, and it will be three stories in height.

---

The Buffalo State Hospital has issued its thirty-second annual report, covering the year ending September 30, 1902. This is in reality the superintendent's report, as the board of managers was wiped out by legislative enactment last year. Dr. Hurd has done himself and the cause which he serves distinguished credit in his management of this great institution. He is surrounded by an able staff and the whole working of the state hospital is a matter of pride to the citizens of Buffalo. This report contains an obituary notice of Dr. Thomas Lothrop, who served on the board of managers for ten years. When the board was abolished a board of visitation was created to which Dr. Lothrop was appointed. Upon his death Dr. William C. Krauss was designated to fill the vacancy. The total number of patients treated during the fiscal year was 2,296, of which 1,059 were men and 1,237 were women. The population of the hospital has steadily increased year by year until the limit of its capacity is practically reached. It, therefore, will become necessary for the state to consider the propriety of a further enlargement of the institution.

---

THE Riverside Accident Hospital was recently transferred from the Fitch Institute to more commodious quarters, at 118 Swan street, Buffalo, where it is installed in a three-story brick building, more accessible and better adapted to its needs.

---

DR. T. D. CROTHERS, of Hartford, professor of mental and nervous diseases at the New York School of Clinical Medicine, will deliver a course of four lectures on Inebriety from alcohol, opium and other narcotics, January 5 and 6, 1904, at 4 p. m. and 8 p. m., at the school, 328 West Forty-second street. The public are cordially invited to attend.

---

A NEW hospital is projected at Bradford, Pa., to be built on a hillside, constructed of stone and brick, and will have the most approved equipment. The plans were drawn by Green & Wicks, the Buffalo architects, and the chairman of the building committee is Dr. James Johnston, of Bradford.

